# Vulval Fibroadenoma — A Report of Two Cases with Review of Literature

**Published:** 2014-06

**Authors:** R. Kalyani, Murthy V. Srinivas, P. Veda

**Affiliations:** Department of Pathology, ESIC Medical College and PGIMSR, Rajajinagar, Bangalore, Karnataka, India

**Keywords:** Ectopic breast tissue, fibroadenoma, vulva

## Abstract

Vulval fibroadenoma is rare benign tumours arising from ectopic breast tissue or mammary like anogenital glands tissue. Only a few cases are reported in medical literature. It is usually seen between 20 – 80 years of age. Excision usually has good prognosis and rarely recurs. We present two cases of vulval fibroadenoma, one in a 26 years woman as a well defined soft tissue mass in right labia major and other in a 45 years woman as a pedunculated soft tissue mass in left labia major.

## INTRODUCTION

Vulval fibroadenoma is a mammary like fibroepithelial lesion of uncertain histogenesis. It is infrequent and extremely rare having low incidence. It is sporadically reported. Vulval fibroadenoma is reported since past 50 years in medical literature (1-4). Till date 50 cases of ectopic benign breast lesions and 20 cases of ectopic malignant breast lesions have been reported in the literature (3). It is seen between 20–80 years of age, usually after puberty and enlarges gradually (1, 2, 4). The histogenesis of vulval fibroadenoma is controversial. The clinical presentation, histopathological features and prognosis is similar to that of breast fibroadenoma. We present two cases of vulval fibroadenoma, one in a 26 years woman and other in a 45 years woman.

## CASE REPORT 1

**Figure 1:**
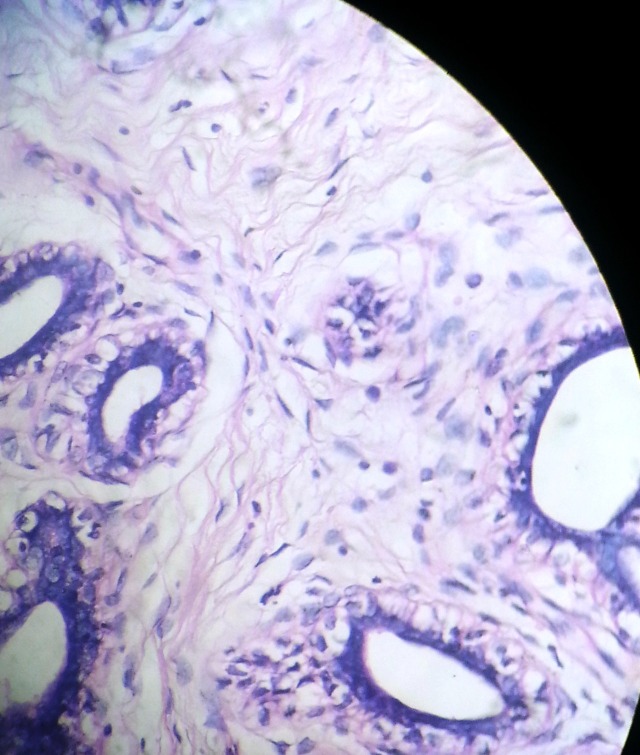
Microphotograph showing intracanalicular and pericanalicular pattern of fibroadenoma. H&E ×100.

A 26 years woman presented with a painless, well defined mass in right labia major since one year, gradually increasing in size. A clinical provisional diagnosis of fibrolipoma was made. Fine needle aspiration cytology was done and a diagnosis of benign adnexal tumour was made. The excised specimen grossly was a grey white nodular mass of 4.5 × 2.4 × 1.2 cms covered partially by elliptical bit of skin measuring 2 × 0.5 cms. Cut section was solid grey white and lobulated. Microscopy showed proliferating fibrous stroma in a peri and intracanalicular pattern. Some ducts were cystically dilated and others compressed into slit like spaces. The ducts were lined by bilayered epithelium, inner columnar to cuboidal and abluminal the myoepithelial cells (Fig. 1). Histopathologically a diagnosis of vulval fibroadenoma was made. The patient was followed for one year and it was uneventful.

## CASE REPORT 2

**Figure 2:**
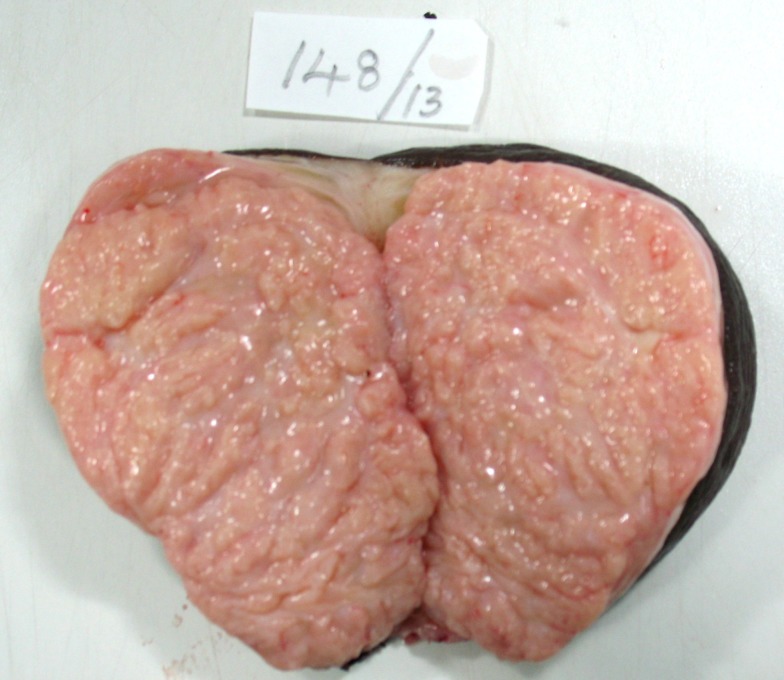
Gross photograph showing cut surface of oval gray white soft tissue with slit like areas and externally completely covered by skin.

**Figure 3:**
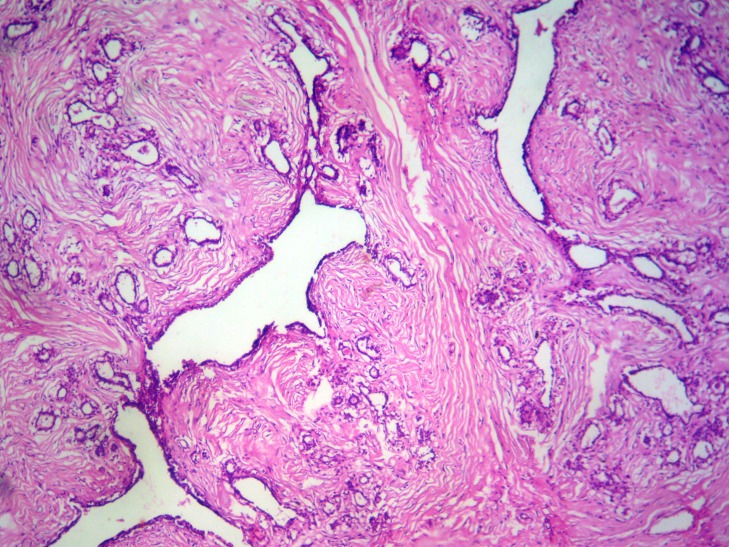
Microphotograph showing branching tubular glands, some dilated and others compressed by stroma. H&E ×100.

A 45 years woman presented as a pedunculated mass in left labia major since eight months. A provisional clinical diagnosis of vulval lipoma was made. The excised specimen grossly showed a pedunculated mass, completely covered with skin and measured 12 × 8 × 6.5 cms. Cut surface was grey white with slit like areas (Fig. 2). Microscopy showed features of fibroadenoma (Fig. 3). The patient was followed for 10 months without any recurrence.

## DISCUSSION

Hartung presented the first description of vulval fully formed mammary tissue in 1872. Bardsley Petterson described 13 cases of vulval mammary tissue originated primary breast carcinoma in the literature. Yin et al described first case of ectopic mammary tissue mucinous adenocarcinoma in vulva. The ectopic or aberrant mammary tissue occurs in 1-6% of the population (2, 3, 5). The accessory mammary tissue presenting as polymasia has been reported in 5% Japanese women. Majority has one additional breast but up to ten additional breasts have been reported. Accessory breast has several forms and has been classified by Kajava as follows: 1) Complete breast with nipple, areola and glandular tissue; 2) Supernumery breast without areola but with nipple and glandular tissue; 3) Supernumery breast without nipple but with areola and glandular tissue; 4) Aberrant glandular tissue without nipple or areola; 5) Pseudomamma with nipple and areola but without glandular tissue; 6) Polythelia, presence of nipple only; 7) Polythelia areolaris, presence of areola only; 8) Polythelia pilosa, presence of only patch of hair. Some aberrant breast tissue is also reported outside milk line as shoulders, buttocks or ears which reflect a reversion to characteristics of lower animals. Breast tissue is normally present in vulva in Whales, Porpoises and in Dolphins (5).

The histogenesis of vulval fibroadenoma is controversial, debatable and uncertain. Two theories have been put forth. The first theory states that vulval fibroadenoma arises from ectopic mammary tissue derived from primitive embryological milk line. Milk line appears in 5th week of gestation when the embryo is 7 mm length and extends from axilla to medial aspect of groin. Soon the milk line involutes except in mid-thoracic region where it form band like epidermal thickening and develops as mammary ridge and then into breast. Incomplete involution of the mammary streak gives rise to ectopic mammary tissue along embryonic milk line. The vulval ectopic mammary tissue is formed from inferior end of primitive mammary streak. The theory of milk ridge is proposed in early years of 19th century and was stated that the theory could be applied to humans (1-4). The second recent / alternative theory is put forth by Putte in 1994 and he criticized the theory of milk ridge stating it to be out of question, not proved definitely and it will not explain perineal ectopic breast tumours. He suggested the presence of specialised glands identical to mammary glands which exist in the anogenital area normally and has close relationship with eccrine glands. These glands are called mammary like anogenital glands which is present in vulva. These were also thought to be adnexal glands like mammary glands. Putte’s theory also states that the existence of normal breast tissue is not necessary for the foundation of these lesions (2-5).

The ectopic breast tissue or mammary like anogenital glands tissue express hormone receptors which are detected by immunohistochemistry and are potential to present with benign or malignant lesion similar to normal mammary tissue and also with lactational changes. The role of increased serum estradiol over progesterone in the pathogenesis as in breast fibroadenoma needs further evaluation (2, 4).

The vulval fibroadenoma is seen between 20–80 years of age and presents as cutaneous/subcutaneous nodules, usually solitary on labia major, with sharp borders, rarely as vulval cysts and seldom bilateral similar to those in the breast. It can present as painful or asymptomatic swelling. The swelling grow significantly during pregnancy and lactation (1-4). The differential diagnosis can be either benign or malignant lesions. The benign lesion like epidermal cyst, follicular cyst, Bartholin’s gland duct cyst, lipoma, hidradenoma papilliferum, lactating adenoma, intraductal papilloma, apocrine adenoma, phyllodes, syringoma, pseudoangiomatous stromal hyperplasia, fibrocystic disease and sclerosing adenosis. Malignant lesions which are differential diagnosis for vulval fibroadenoma are extramammary Paget’s disease, ductal/lobular/mucinous adenocarcinoma (1-5). In our cases, the first case was seen in 26 years woman as a subcutaneous solitary well defined soft tissue painless mass and the provisional clinical diagnosis was vulval fibrolipoma. Second case was seen in 45 years woman as a pedunculated soft tissue mass with provisional clinical diagnosis of vulval lipoma.

The vulval fibroadenomas are diagnosed by fine needle aspiration cytology (FNAC) and histopathological examination of the tissue. The lesion usually measures 0.8 to 6.0 cms in size. Histopathological features are similar to those in breast, having tubular branching/compressed glands with fibromyxoid stroma surrounding it. The glands are lined by luminal columnar cells, some showing apocrine change and abluminal myoepithelial cells. The tumour usually has a definite capsule. The presence of normal ductular structures adjacent to the lesion indicates its origin from ectopic breast tissue (1, 2). The tumour is positive for ER, PR, SMA, S100, CK, EMA and GCDF-15 by immunohistochemistry. However expression of these receptors is not specific for tumours of mammary origin (1, 4). In our cases, in first case FNAC was done and a cytological diagnosis of benign adnexal tumour was made. In second case the lesion measured 12 × 8 × 6.5 cms. The histopathological features in both cases showed classical features of fibroadenoma. Both cases were positive for ER and PR markers by immunohistochemistry.

The behaviour of the tumour is similar to that in the breast. Excision usually has good prognosis and rarely recurs (2). In our cases, both were uneventful when followed for one year in first case and ten months in second case.

To conclude, this rare cases of vulval fibroadenoma is reported to increase the number of cases reported in the literature and to bring the awareness of vulval ectopic breast tissue/mammary like anogenital glands. In the second case the presentation as a pedunculated soft tissue mass is an unusual presentation.
